# Utility of Multi-Gene Loci for Forensic Species Diagnosis of Blowflies

**DOI:** 10.1673/031.011.5901

**Published:** 2011-05-05

**Authors:** Farrah Zaidi, Shu-jun Wei, Min Shi, Xue-xin Chen

**Affiliations:** ^1^ institute of Insect Sciences, Zhejiang University, Hangzhou 3 10029, China; ^2^ Department of Zoology, University of Peshawar, Peshawar 25120, Pakistan

**Keywords:** Calliphoridae, cytochrome b, molecular identification, NADH dehydrogenase 5, nuclear internal transcribed spacers

## Abstract

Contemporary studies in forensic entomology exhaustively evaluate gene sequences because these constitute the fastest and most accurate method of species identification. For this purpose single gene segments, cytochrome oxidase subunit I (COI) in particular, are commonly used. However, the limitation of such sequences in identification, especially of closely related species and populations, demand a multi-gene approach. But this raises the question of which group of genes can best fulfill the identification task? In this context the utility of five gene segments was explored among blowfly species from two distinct geographic regions, China and Pakistan. COI, cytochrome b (CYTB), NADH dehydrogenase 5 (ND5), nuclear internal transcribed spacers (ITS1 and ITS2), were sequenced for eight blowfly species including *Chrysomya megacephala* F. (Diptera: Calliphoidae), *Ch. pinguis* Walker, *Lucilia sericata* Meigen *L. porphyrina* Walker, *L. illustris* Meigen *Hemipyrellia ligurriens* Wiedemann, *Aldrichina grahami* Aldrich, and the housefly, *Musca domestica* L. (Muscidae), from Hangzhou, China; while COI, CYTB, and ITS2 were sequenced for four species, i.e. *Ch. megacephala, Ch. rufifacies, L. cuprina*, and the flesh fly, *Sarcophaga albiceps* Meigen (Sarcophagidae), from Dera Ismail Khan Pakistan. The results demonstrate a universal utility of these gene segments in the molecular identification of flies of forensic importance.

## Introduction

Blowflies are the first insects to arrive at the scene of death, often seen ovipositing on the cadaver during the first few hours after death (Smith 1986). The growth periods, developmental rates (van Laerhoven 2008), and diapause responses (Ash and Greenberg 1975) are all substantially different for closely related species. These peculiar features make blowflies the primary and most accurate indicators of the post mortem interval (Grassberger et al. 2003). However, the immature stages pose a great identification challenge for forensic entomologists due to their lack of species-specific anatomical characters (McDonagh et al. 2009). Identification of these stages using traditional methods (Mendonca et al. 2008) and/ or advanced techniques (McDonagh et al. 2009) is therefore the focus of many forensic studies. The most popular method of identification in recent years includes molecular taxonomy (Harvey et al. 2003; Smith and Baker 2008). In this regard several loci have been explored for their phylogenetic utility, but mitochondrial cytochrome oxidase subunit I (COI) (Harvey et al. 2003; Ratcliffe et al. 2003; Nelson et al. 2007; Ying et al. 2007; Smith and Baker 2008) is often used, sometimes along with subunit II (COII) (Ratcliffe et al. 2003; Ying et al., 2007). The 5′ end of COI is also the site of the proposed universal animal DNA “barcode” (Hebert et al. 2003). COI barcodes have been successfully utilized in the identification of many blowfly species (Nelson et al. 2007). However, the barcoding approach has its own limitations (Moritz and Cicero 2004) as is seen with its failure to identify some closely related species of blowflies (Nelson et al. 2007; Whitworth et al. 2007). Thus, COI barcodes, or for that matter any single gene, seems unlikely to resolve the identities of all calliphorid species of forensic importance because such phylogenies only infer the evolutionary relationships for the particular gene used. Consequently, a switch to multigene approach becomes necessary (McDonagh et al. 2009). To date only a handful studies have utilized such an approach (Wallman et al. 2005).

**Table 1.**
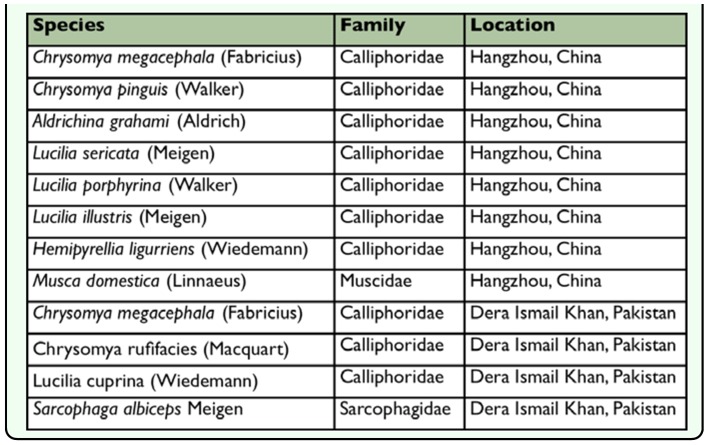
Species collected from Hangzhou, China and Dera Ismail Khan, Pakistan

Therefore, a multi-gene approach was employed for the identification of blowfly species of forensic importance from Hangzhou, China and Dera Ismail Khan, Pakistan. Gene data was freshly generated for a novel combination of five loci, including the barcode region of mitochondrial cytochrome oxidase subunit I (COI) (Hebert et al. 2003), NADH dehydrogenase 5 (ND5), cytochrome b (CYTB), and nuclear internal transcribed spacers 1 and 2 (ITS1 and ITS2).

## Materials and Methods

### Sample collection and Identification

Samples of blowflies were collected from Huajiachi, Hangzhou over the period March 2007 to June 2008, while in Dera Ismail Khan sampling was carried out in December 2008. Identification was performed using morphological keys (Fan 1992; Fan 1997; Senior-White et al. 1940). The identified species are listed in Table 1.

**Table 2.**
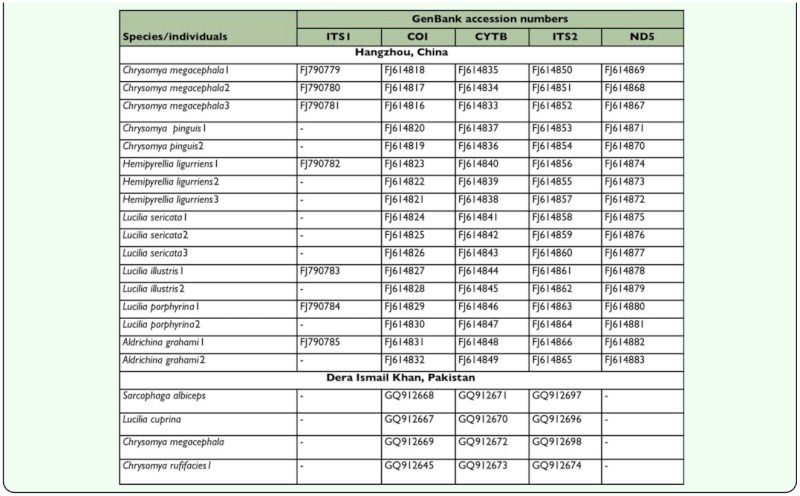
New sequences of flies from Hangzhou, China and Dera Ismail Khan, Pakistan

### DNA extraction, amplification and sequencing

Twenty-six individuals including 22 blowflies (7 species) from Hangzhou and 4 individuals (including 4 species) from Dera Ismail Khan (Table 1) were used for DNA extraction, amplification, and sequencing. Total DNA was extracted from leg and thorax regions of each adult fly using the DNeasy Tissue Kit (QIAGEN, www.qiagen.com) following the manufacture's protocol. Previously reported primers along with protocols of polymerase chain reaction (PCR) conditions were used to amplify the COI, ITS2 (Nelson et al. 2007), ND5 (Zehner et al., 2004), ITS1, and CYTB (Kengne et al. 2007). Thermal cycling was performed in a Mastercycler (Eppendorf, www.eppendorf.com). Amplified products were purified and sequenced by Shanghai Invitrogen (www.invitrogen.com). The newly sequenced blowfly specimens from Hangzhou and Dera Ismail Khan with their related GenBank accession numbers for ITS1, COI, ITS2, ND5, and CYTB are listed in Table 2. The previously published sequences utilized during the present study are presented in Table 3.

### Sequence analysis

All sequences were partial except for ITS2. Each COI, CYTB, and ND5 sequence represents part of the corresponding genes of *M. domestica* (GenBank accession EU 154477) with COI pertaining to positions 1385–2040 (655 bp), CYTB to 10722–11192 (470 bp), and ND5 to 6340–6785 (445 bp) while ITS-II with its 422 bp length was completely sequenced. ITS1 sequences were not used in the phylogenetic analysis due to the difficulties in alignment as well as an inadequate data set. Nevertheless, ITS1 was sequenced for most of the species from Hangzhou with the legible length ranging between 400 to 700 bp.

**Table 3.**
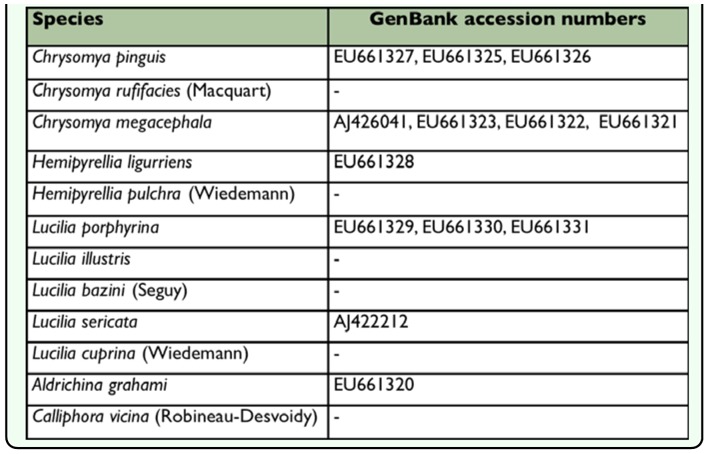
Previously published COI sequences used in our phylogenetic analysis

The software Dnadist (Felsenstein 1989) was used to compute distance matrices for nucleotide sequences of different gene segments and graphs were plotted using the same data.

Chromas Pro 34-Version 1.33 (available online, www.technelysium.com.au/ChromasPro.html was used for manual editing of sequences that were subsequently aligned in CLUSTAL X using default parameter settings.

Phylogenetic analyses were performed using maximum parsimony (MP) with PAUP* 4.0b10 (Swofford 2002) and maximum likelihood (ML) with PhyML (Guindon et al. 2005). The MP analyses were run with default heuristic search options except that 100 replicates of random stepwise additions were used. Models of DNA substitution were estimated in Modeltest 3.7 (Posada and Crandall 1998). For ML we used GTR + G model for COI, Cytb and ND5, and F81+G model for ITSII nucleotide sequences. Bootstrap proportions were obtained after 1000 replicates by using 10 replicates of random stepwise additions of taxa.

**Table 4.**
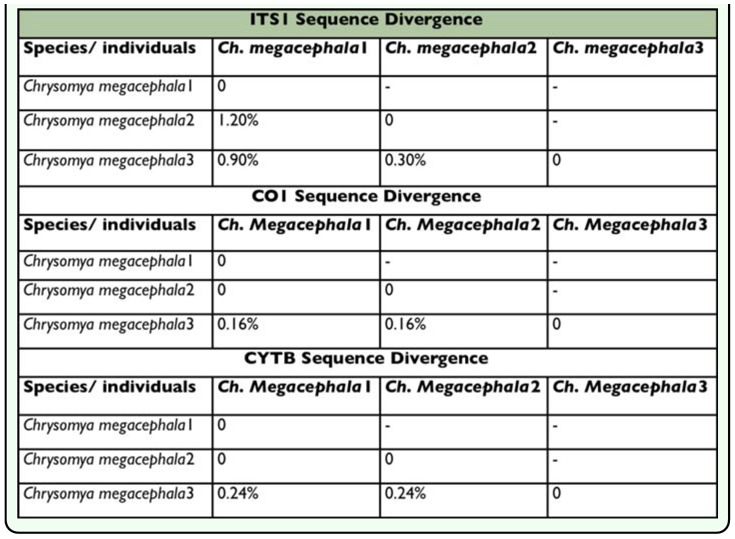
Intra-specific ITSI, COI and CYTB divergence among *Chiysomya megacephala* individuals

## Results and Discussion

Sequence data were generated for 12 species, including 10 blowflies, from two geographic zones of Pakistan and China.

### Sequence variability for ITS

The transcribed spacer ITS2 has been sequenced recently and successfully utilized in the molecular identification of blowflies (Nelson et al. 2007; Song et al. 2008). However, ITS1 has not been sequenced before for blowflies. This region was found to be highly variable and was amplified with great difficulty. The species *L. sericata, L. cuprina, Ch. rufifacies*, and *S. albiceps* posed difficulties in amplification. Additionally, the baseline noise in chromatograms made the ITS1 sequences unreliable for *Ch. pinguis*. In contrast, ITS2 was sequenced without any problems.

### Intra-specific sequence variation

The ITS1 intra-specific sequence divergence was computed only for *Ch. megacephala*. The variation was in a reasonable range, i.e. 0.29 – 1.16 %, and was comparable to the mitochondrial segments of COI and CYTB genes (Table 4). However, previous studies usually showed no intra-specific variation in the Drosophilidae for ITS1 region (Baffi and Ceron 2002).

The ITS2 region showed no intra-specific variation for most of the blowfly species except for *L. sericata*. This result seems to be consistent with an earlier study that suggested that ITS2 cannot be utilized in differentiation of geographical populations of some blowfly species (Song et al. 2008).

ITS1 may be a good tool for identification at the population level. The most important reason is the high mutation rate that cause deletions or insertions. A prominent indel characteristic (a 184 bp long deletion) was detected in one of the *Ch. megacephala* sequences (FJ790781). The size and location suggested a loop deletion. Loop deletions, when present in coding regions, might have deleterious effects on the organism like any other mutation (Stefanovic et al. 2007), but their exact impact on non-coding regions such as ITS is unknown. In any case, use of indels (i.e. insertions and or deletions) as genetic markers has been recommended in phylogenetic studies of natural populations (Väli et al. 2008). Thus a high expectancy of insertion/deletions among ITS1 sequences might make them useful in identification of geographic populations of blowflies of forensic importance.

**Figure 1.**
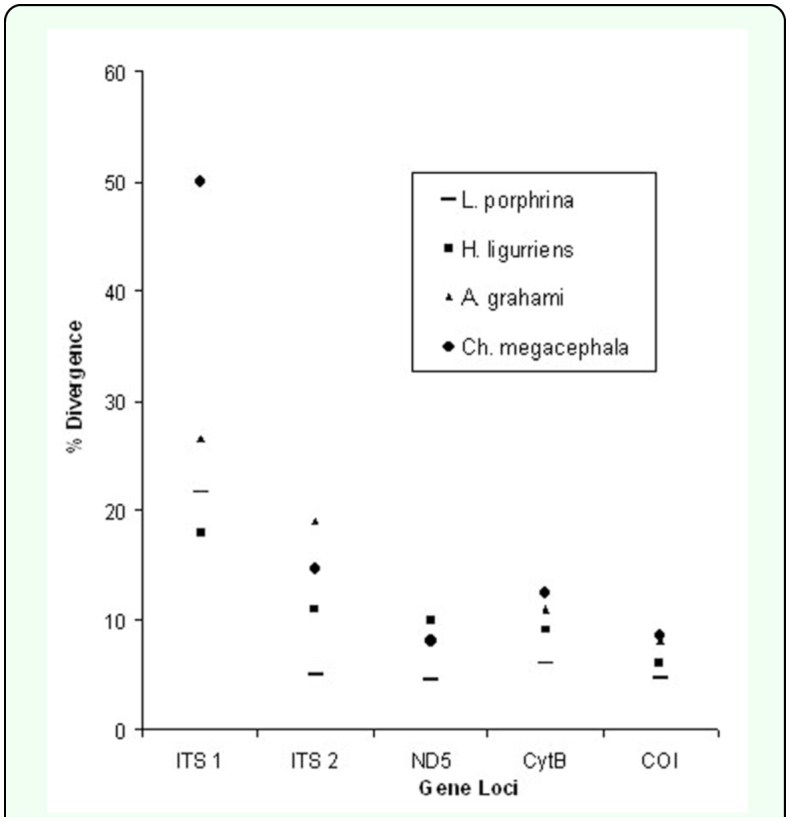
Molecular variation at five loci for *Lucilia illustris* and other blowfly species.Note: *Lucilia illustris* is highly divergent from its sister species *Lucilia porphyrina* (-) at ITSI locus. High quality figures are available online.

### Inter-specific sequence variation

Inter-specific variation was explored for a wide array of paired species (blowfly-blowfly; blowfly-fleshfly, and blowfly-housefly). Both of the ITS regions were found highly variable as compared to mtDNA. The ITS1 segment was abnormally divergent among all gene segments (Supplement Table S1), ranging between 7.3–7.8 % (Figure 1). The results of the present study are consistent with previous studies of Dipteran families, such as Tephritidae, which also revealed little evidence of similarity between ITS1 sequences of species, especially those belonging to different genera (Douglas and Haymer 2001). The most likely reason seems to be the rapidly evolving and mutating nature of non-coding regions, such as ITS (Haymer 1994). On the other hand, ITS1 showed little variation among members of the same species as shown by *Ch. megacephala* individuals (Table 4). These results also showed no overlapping between intra- and inter-specific variations for ITS1. The ITS1 intra-specific variation as recorded for *Ch. megacephala* during the present study was 0.24 – 1.16 % (Table 4), while inter-specific variation between different species pairs was in the range of 8 – 60 % (Supplement Table S1). However, intra- and inter-specific overlapping seems plausible for ITS2 when the smallest inter-specific ITS2 value of divergence is recorded at 1.2 % for *Ch. megacephala* and *Ch. pinguis* (Supplement Table S1). Similar overlapping is previously observed among blowflies (Song et al. 2008), though without any taxonomic impact in that study, nevertheless overlapping can negatively influence the identification process. However, at the family level ITS2 possessed strong resolution power as it showed higher values of variation between blowfly-flesh fly pairs (14.7–21.1 %) and blowfly-housefly pairs (3035.2 %), as compared to blowfly-blowfly pairs (Supplement Table S1). This result is also consistent with previous studies (Song et al. 2008). Another important finding of the present study was abnormally high ITS1 divergence values for sister species *L. porphyrina* and *L. illustris*, i.e. 22 % (Figure 1). At other gene loci, including ITS2, the divergence values between these two species were comparatively low, but adequate for differentiation (4–6 %, Figure 1).

**Figure 2.**
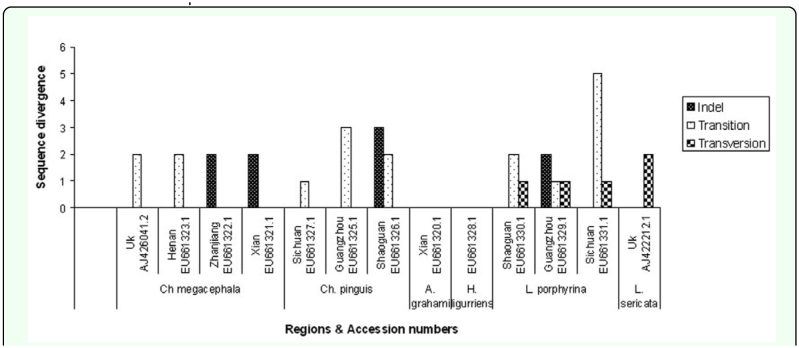
Intra-specific ND5 sequence variation between Hangzhou and other regions. Note: Transitions are the most prominent type of mutations among ND5 sequences. High quality figures are available online.

### Sequence variability for mt DNA

Mitochondrial gene segments showed less sequence variability as compared to the ITS regions although sequence divergence values were almost comparable between CYTB and ITS2 (Figure 1). Insertion and deletions were comparatively fewer in mtDNA genes.

### Intra-specific sequence variation

These results showed a mean of 0.15% COI intra-specific variation for *Ch. megacephala*. The results are similar to those for *Chrysomya* species of blowflies (0.097 %) reported by Nelson et al. (2007). The mt gene CYTB showed variation of 0.24 % among both *Ch. megacephala* and *L. sericata* individuals. On the other hand, ND5 sequences of blowflies were highly conserved with no intra-specific variation. However, comparisons with ND5 sequences from other regions of China that recently became publicly available (Table 3) showed significant variation among the transition/transversion ratios and the indel characteristics between species from Hangzhou and their counterparts in other Chinese cities (Figure 2). These results show the presence of distinct geographic populations of blowflies or perhaps cryptic species. Mutations such as insertions, deletions, transitions, and transversions (Figure 2) are the result of, or an indicator of, divergence of natural populations, making them an important tool in the population identification studies (Väli et al. 2008).

### Inter-specific sequence variation

The inter-specific variation for mtDNA segments, including COI, CYTB, and ND5, was found suitable for blowfly species diagnosis. However, for some sister species this variation was considerably low, e.g. 0.78 % of COI (*L. sericata* vs *L. cuprina*) and 0.5 % of CYTB (*L. sericata* vs *L. cuprina*). Even ITS2 that provided a least variation of 3.5 % for the *L. sericata* and *L. cuprina* pair showed low variation for another blowfly species pair, i.e. *Ch. megacephala* and *Ch. pinguis* (1.2 %). These results justify the need of a novel molecular marker for sister species diagnosis.

**Figure 3.**
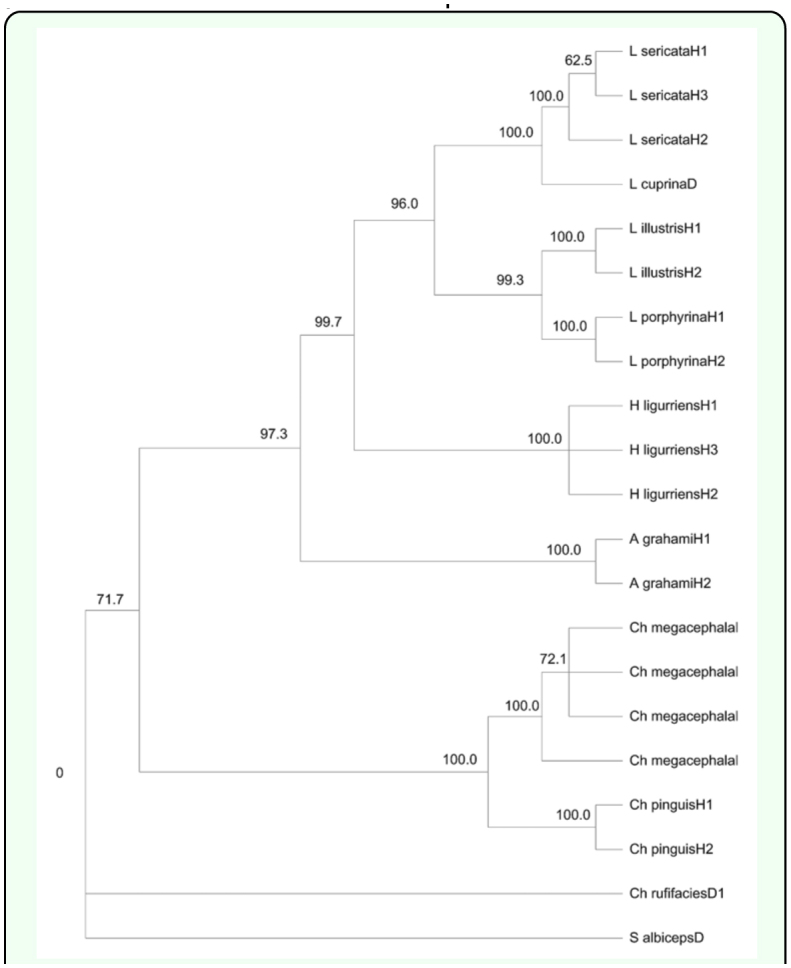
Maximum-parsimony phylogram based on COI (A), CYTB (B), and ITS2 (C) sequences of blowflies from Dera Ismail Khan, Pakistan and Hangzhou, China. High quality figures are available online.

### Phylogenetic analyses

**Figure 4.**
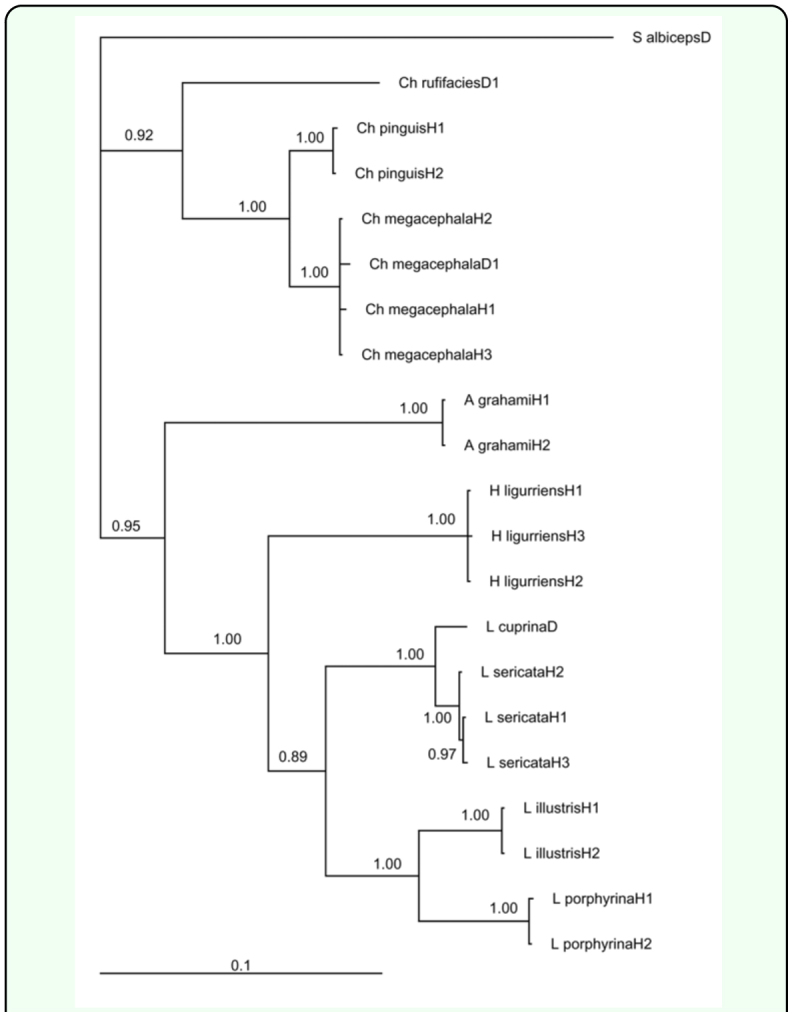
Maximum parsimony (A) and maximum likelihood (B) phylograms based on COI, CYTB, and ITS2 sequences of blowflies from Dera Ismail Khan, Pakistan and Hangzhou, China. High quality figures are available online.

**Figure 5.**
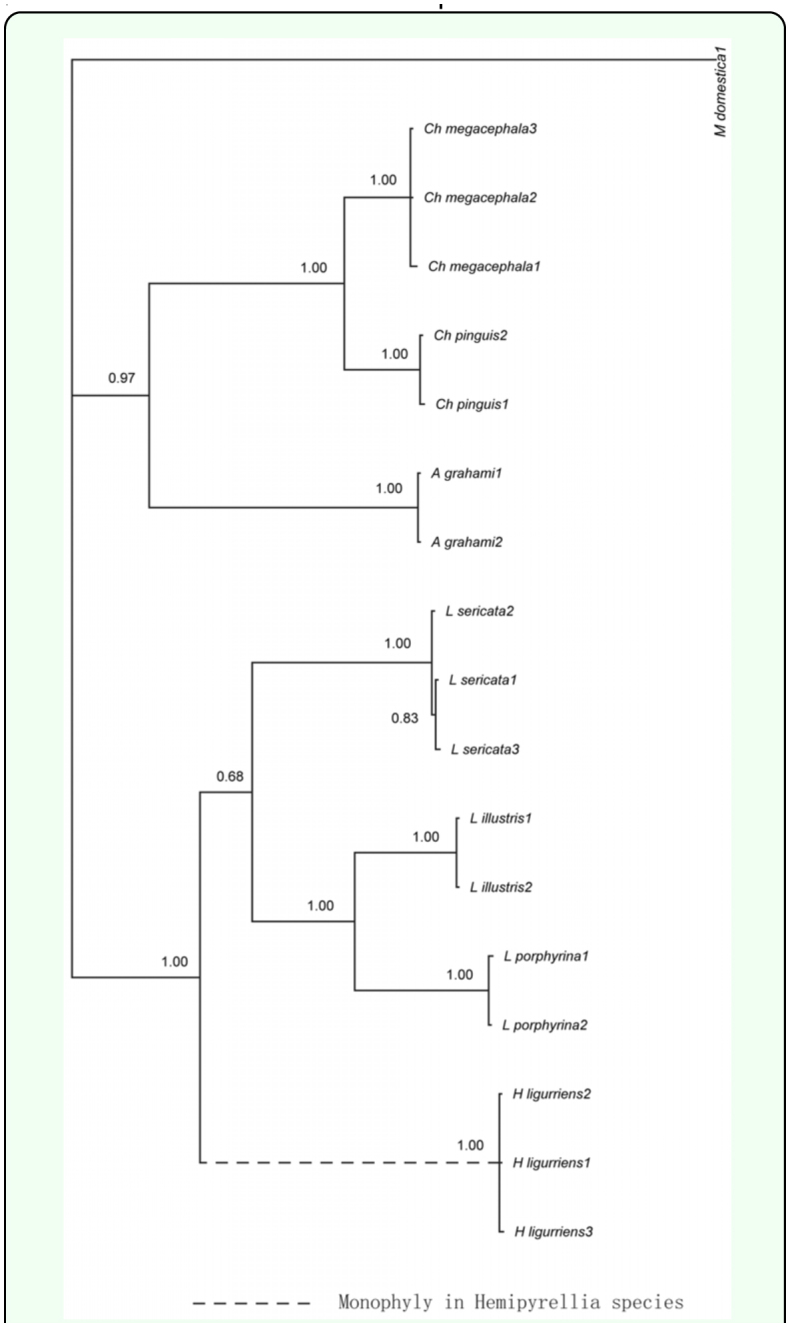
Maximum-likelihood phylogram based on COI, CYTB, ND5, and ITS2 sequences of blowflies. High quality figures are available online.

In an attempt to compare blowfly species from two distinct climatic zones, single (Figures 3A, B, C) and multi-gene trees (Figures 4A, B; Figure 5) based on COI, CYTB, ND5, and ITS2 sequences were constructed utilizing maximum parsimony and maximum likelihood methods. Both approaches identified the 11 fly species of forensic importance from China and Pakistan. This is the first time that the molecular data from the regions of Dera Ismail Khan, Pakistan and Hangzhou, China are presented and compared for blowflies.

### Single vs. multi-gene approach

The significance of single genes in molecular forensics is undeniable (Nelson et al. 2007; Wells et al. 2007; Song et al. 2008). Nonetheless, additional genes become essential in challenging identifications (Nelson et al. 2007). A reasonable group of genes not only clarify doubts and provides validation, but also shed light on new evolutionary relationships. A unique instance is that of *A. grahami*. Grouped together with Lucilini tribe in the COI gene tree (Figure 3A) this species presented a deviation from traditional taxonomy because this species (Calliphorinae) is identified as sisters with Chrysomyiinae rather than Luciliinae (Rognes 1997). Contrary to COI, the CYTB and ITS2 trees (Figures 3b, c) followed this classification. These two distinct patterns of evolution were also observed previously for blowfly phylogenies based on 28rRNA sequences (Stevens and Walls 2001). The multi-gene trees validated both views. The 4-gene ML tree (Figure 5) agreed with the classical taxonomy while the 3-gene MP and ML trees (Figures 4A, B) displayed the alternative scheme.

However, each of the single gene trees, although they adequately identified blowflies, they also showed some limitations in terms of evolutionary relationships between species. For example, the ITS2 tree incorrectly put *H. ligurriens* within genus *Lucilia* (Figure 3C). Previously another single gene phylogeny based on the COI locus also grouped this species with *L. cuprina* (Wells et al. 2007). The multi-gene trees made amendments by supporting the monophyletic status of genus *Hemipyrellia* (Figures 4A, B). *Chrysomya rufifacies* provided another example of misplacement by single gene tree, CYTB in this case (Figure 3B). The 3-gene ML tree clarified this ambiguity by rightly placing this species along with other species of genus *Chrysomya* (Figure 4B).

These results demonstrate a universal utility of the respective five gene segments. Though single genes exhibited easy identification, the wrong placing of some species illustrates the possibility of future misdiagnosis. Therefore a switch to a multi-gene tactic was made with future work aiming at the examination of more variable segments and species from distinct and multiple geographic regions.

## Abbreviations:

**COI**,: cytochrome oxidase subunit I;
**CYTB**,: cytochrome b;
**MP**,: maximum parsimony;
**ML**,: maximum likelihood;
ITS1, ITS2,: nuclear internal transcribed spacers 1 and 2;
ND5,: NADH dehydrogenase 5

## Supplement Table S1

**Supplement Table S1.** Pair-wise comparison of inter-specific variation among different gene segments
