# Supplementary material for: Utility of Multi-Gene Loci for Forensic Species Diagnosis of Blowflies
Source: J Insect Sci. 2011 May 5;11:59. doi: 10.1673/031.011.5901 (PMC3391916; doi:10.1673/031.011.5901)
Supplement: Supplementary file 1 [file sd1_1.pdf]

[illegible]

Species	% sequence variation				
	ITS1	ND5	ITS2	COI	9:33 PM
Ch. megacephala vs Ch. pinguis	-	5	1.2	2.2-2.5	5.2- 5.5
Ch. megacephala vs L. illustris	50-35	7.7	14.6-14.9	8.3 -8.5	12.2-12.5
Ch. megacephala vs L. porphyrina	25-27.2	9.1	15.4 -15.7	9.8- 9.9	12-12.2
Ch. megacephala vs L. sericata	-	10.3	15.8- 16.4	9.1-9.2	14 -14.5
Ch. megacephala vs. H. ligurriens	28-31.2	10.9	17	8.3-8.65	15.1- 15.4
Ch. megacephala vs. A. grahami	51-57.3	10.4	8.7	8.5-8.6	12.7-13
Ch. megacephala vs. M. domestica	-	16.3	30.2-30.4	10.3-10.5	15.1- 15.4
Ch. pinguis vs. L. Illustris	-	9.7	14.4	9.4-9.5	13.4
Ch. pinguis vs. L. porphyrina	-	9.9	15.7	10.3-10.4	11.7
Ch. pinguis vs. L. sericata	-	10.8	15.6-16	10 -10.1	13-13.1
Ch. pinguis vs. H. ligurriens	-	12.1	16.3	8.8 - 9.2	14
Ch. pinguis vs A. grahami	-	10.6	8.8	8.8-9	12.5
Ch. pinguis vs. M. domestica	-	17	30.2	11-11.2	16.7
Ch. rufifacies vs. Ch. megacephala	-	-	7.5-7.7	6.5- 6.8	13.7-13.9
Ch. rufifacies vs. Ch. pinguis	-	-	6.8	8-8.2	11.9
Ch. rufifacies vs. L. sericata	-	-	16.2-16.4	11.6	12.7-13
Ch. rufifacies vs. L. illustris	-	-	17	10.8	11.2
Ch. rufifacies vs. L. porphyrina	-	-	15.8	11.95-12.1	14.7
Ch. rufifacies vs. H. ligurriens	-	-	18.9	10.4-10.6	9
Ch. rufifacies vs. A. grahami	-	-	8.7	9.4	11.7
Ch. rufifacies vs. S. albiceps	-	-	15.4	10.3	12.4
Ch. rufifacies vs. M. domestica	-	-	31	12.4	15
L. illustris vs. L. porphyrina	22	4.5	5.1	4.6 (4)	6.1
L. illustris vs. L. sericata	-	7.9	11.1-11.4	4.7	9.1
L. illustris vs. H. ligurriens	17.7	10.1	10.6	6 -6.2	9
L. illustris vs. A. grahami	26.6	7.8	19	8.5	10.6
L. illustris vs. M. domestica	-	16.2	30	10.9	12.5
L. porphyrina vs. L. sericata	-	8.1	11.6-12	5.8-6	8
L. porphyrina vs. H. ligurriens	7.8	10.1	11.4	7.2-7.6	10.4
L. porphyrina vs. A. grahami	41.3	8.1	17.7	8.8-9	11.8
L. porphyrina vs. M. domestica	-	16.5	33	12.5	13.7
L. sericata vs. L. cuprina	-	-	3.5-3.7	0.78	0.5-0.7
L. sericata vs. H. ligurriens	-	9.5	13.7-14	5.2-5.4	9.5-9.8
L. sericata vs. A. grahami	-	10.4	18.4-18.7	7.6	13.2-13.5
L. sericata vs. M. domestica	-	16.3	33.4-33.8	12.4	16.4
L. cuprina vs. L. Illustris	-	-	11.3	4.8	9.1
L. cuprina vs. L. porphyrina	-	-	12.2	5.8	8.5
L. cuprina vs. H. ligurriens	-	-	14	5.5-5.7	9.6
L. cuprina vs. A. grahami	-	-	16.2	7.2	13.2
L. cuprina vs. Ch. megacephala	-	-	14-14.6	8.8-9.2	14.6-14.9
L. cuprina vs. Ch. pinguis	-	-	14.4	10.1-10.3	13.7
L. cuprina vs. Ch. rufifacies	-	-	15	10.9-11.1	12.2
L. cuprina vs. S. albiceps	-	-	19.5	10.1	16
L. cuprina vs. M. domestica	-	-	32.5	12.6	16.5
H. ligurriens vs. A. grahami	42.8	12.4	18.7	7.4 -7.6	10.4
H. ligurriens vs. M. domestica	-	16	30	12.1-12.4	13.5
A. grahami vs. M. domestica	-	16.3	35.2	13.5	15.5
S. albiceps vs. Ch. megacephala	-	-	14.7-15	9.9-10.3	15.9-16.2
S. albiceps vs. Ch. pinguis	-	-	14.8	10.7-10.8	16.2
S. albiceps vs. L. sericata	-	-	19-19.3	10.5	16
S. albiceps vs. L. illustris	-	-	18.1	10.3	14.5
S. albiceps vs. L. porphyrina	-	-	18.3	12.3-12.5	15.4
S. albiceps vs. H. ligguriens	-	-	21.1	11.8 -12	14
S. albiceps vs. A. grahami	-	-	15.8	11.6	17 (2)
S. albiceps vs. M. domestica	-	-	29.5	13.5	12.7
Variation at species level (blowflies vs. blowflies)	7.8– 57.3 %	4.5– 12.4 %	1.2– 19 %	0.78– 10.4 %	0.5– 15.4 %
Variation at family level (blowflies vs. houseflies & blowflies vs. flesh flies)	-	16– 17 %	14.7– 35.2 %	9.9– 13.5 %	12.5– 16.7 %
